# Data augmentation based on multiple oversampling fusion for medical image segmentation

**DOI:** 10.1371/journal.pone.0274522

**Published:** 2022-10-18

**Authors:** Liangsheng Wu, Jiajun Zhuang, Weizhao Chen, Yu Tang, Chaojun Hou, Chentong Li, Zhenyu Zhong, Shaoming Luo

**Affiliations:** 1 Academy of Interdisciplinary Studies, Guangdong Polytechnic Normal University, Guangzhou, China; 2 Academy of Contemporary Agriculture Engineering Innovations, Zhongkai University of Agriculture and Engineering, Guangzhou, China; 3 Institute of Intelligent Manufacturing, Guangdong Academy of Sciences, Guangzhou, China; Politechnika Slaska, POLAND

## Abstract

A high-performance medical image segmentation model based on deep learning depends on the availability of large amounts of annotated training data. However, it is not trivial to obtain sufficient annotated medical images. Generally, the small size of most tissue lesions, e.g., pulmonary nodules and liver tumours, could worsen the class imbalance problem in medical image segmentation. In this study, we propose a multidimensional data augmentation method combining affine transform and random oversampling. The training data is first expanded by affine transformation combined with random oversampling to improve the prior data distribution of small objects and the diversity of samples. Secondly, class weight balancing is used to avoid having biased networks since the number of background pixels is much higher than the lesion pixels. The class imbalance problem is solved by utilizing weighted cross-entropy loss function during the training of the CNN model. The LUNA16 and LiTS17 datasets were introduced to evaluate the performance of our works, where four deep neural network models, Mask-RCNN, U-Net, SegNet and DeepLabv3+, were adopted for small tissue lesion segmentation in CT images. In addition, the small tissue segmentation performance of the four different deep learning architectures on both datasets could be greatly improved by incorporating the data augmentation strategy. The best pixelwise segmentation performance for both pulmonary nodules and liver tumours was obtained by the Mask-RCNN model, with DSC values of 0.829 and 0.879, respectively, which were similar to those of state-of-the-art methods.

## Introduction

According to the data released by the Global Burden of Cancer worldwide (GLOBOCAN), cancer is the leading cause of death in the world. Early detection and treatment are the key means to reduce cancer mortality [1]. Through effective treatment of early cancer, the five-year survival rate can be increased to more than 90% and the cure rate can be improved. Computed Tomography (CT) and Magnetic Resonance Imaging (RMI) are non-invasive, painless and accurate technologies for identifying human tissue lesions to help clinical experts diagnose and plan treatment plans. Nowadays, CT is becoming more and more popular in the diagnosis and further treatment of cancer and its progression [2]. For pathological examination of chest and abdomen organs such as heart, lung, liver and gallbladder, the processing performance using CT is better than that using MRI. Moreover, monitoring and analysis using CT images is an important strategy for early cancer diagnosis [3]. Accurate segmentation of the lesion region in the tissue will directly affect the subsequent analysis results [4].

Recently, deep learning in the medical image analysis field has accomplished remarkable achievements—including the recognition and segmentation of lesion tissues based on deep learning [5]. Although deep neural networks have achieved great success in medical image segmentation, the lack of effective annotated data is still a major problem [6]. In addition, the scale of some lesion tissues in CT images is very small, especially in the early stages, resulting in the extreme imbalance of categories in the dataset. This class imbalance in the dataset affects network convergence during the training stage, which adversely affects model performance [7]. Therefore, given the limited data situation, it is critical to conduct research on classification imbalance and small object segmentation methods to ensure accurate segmentation.

To further improve the segmentation performance of deep neural networks in tissue lesions, many scholars have performed much research on networks with different network architectures [8]. These models reached performance levels similar to those of experienced radiologists. Wang et al. [9] proposed a central focus convolution neural network (CF-CNN), which combines two-dimensional and three-dimensional CT images to obtain diverse combinations of nodal features, allowing it to achieve good pulmonary nodule segmentation accuracy from CT images. Its best segmentation result was a Dice similarity coefficient (DSC) of 0.82. Kopelowitz and Engelhard [10] proposed a three-dimensional feature extraction strategy using Mask-RCNN, which can extract three-dimensional pulmonary nodule features from CT images, which are subsequently used to recognize and segment pulmonary nodules. Jin et al. [11] proposed a three-dimensional hybrid residual attention perception segmentation method, RA-UNet, to accurately extract liver interest volume (VOI) and segmented tumours from liver interest volume by introducing a residual learning mechanism to realize the extraction and combination of a low-level feature map and a high-level feature map. Li et al. [12] proposed a new hybrid DenseUNet (H-DenseUNet), which consists of 2D DenseUNet for efficient extraction of on-chip features and a 3D counterpart for layered aggregation volume context in the spirit of automatic context algorithms. The in-chip representation and interchip features can be optimized by a mixed feature fusion (HFF) layer. The liver tumour segmentation performance of the network was tested on the LiTS17 dataset, and a dice global value of 82.4 was obtained, which was better than that of the first place in the LiTS17 competition with a dice global value of 81.3. Although these methods have reached a new height in terms of performance in tissue lesion segmentation, the generalization ability of neural network models is still relatively weak due to the lack of effectively annotated training data.

Class imbalance in datasets is another common problem in deep learning. To solve this problem, Wang et al. [13] proposed a new mean square error loss function that effectively and simultaneously catches classification errors for both the majority and minority classes. This error loss function effectively improved model accuracy on unbalanced datasets. Khan et al. [14] proposed a cost-sensitive deep neural network that jointly optimizes the class-related costs and neural network parameters during training to automatically learn robust features for both the majority and minority classes. This approach improves the classification accuracy without changing the distribution of the original data. Wang et al. [15] developed a novel fine-grained classification method for CT pulmonary nodules that used a generative adversarial network (GAN) to enhance the features of pulmonary nodules and ameliorated data category imbalances. However, training the GAN itself requires considerable training data, generating the trained GAN requires complex iterations, and the model can easily fall into a local optimum. Fortunately, in machine learning, oversampling is the most widely used method to alleviate class imbalance. Mateusz et al. [16] proved that oversampling to alleviate category imbalances is a suitable technique for deep learning. Kisantal et al. [17] proposed a method to improve the network performance in object recognition and segmentation by oversampling small objects. This method improved both the segmentation and recognition accuracy of small objects by 9.7% and 7.1%, respectively, on the common objects in the context (MS COCO) dataset. Yang et al. [18] also proposed a data augmentation method and applied it to small-target segmentation in automatic driving scenes. Continuously oversampling the small-size objects with a boundingbox less than 32 × 32 pixels increases the frequency of small targets in the image, which might lead to an increase in the probability of more accurate small target segmentation. However, these methods are not suitable for target segmentation problems with only small amounts of data samples.

Many scholars have conducted a large number of studies to alleviate category imbalance, but these studies are all conducted with a large number of samples or with large target objects. However, the lack of training data and category imbalance are common problems in medical image segmentation. In this paper, we proposed a new method for data augmentation when data are lacking and the dataset categories are imbalanced. The main contributions are as follows:

1. We propose a data augmentation method combining multiple oversampling and affine transformation values. This method can increase the data diversity of the target object in the data and improve the segmentation accuracy.
2. It is proposed to increase the number of small targets in the same image by means of repeated sampling, which can improve the problem of category imbalance in the data, so as to improve the ability of network segmentation of small targets.
3. By re-training the existing U-Net, SegNet, DeepLabv3 + and Mask-RCNN models on our synthetic datasets, we obtained the best pixelwise segmentation performance for both pulmonary nodules and liver tumours was obtained by the Mask-RCNN model, with DSC values of 0.829 and 0.879, respectively.

## Materials and methods

### Dataset

To evaluate the impact of the proposed data augmentation method on the performance of the deep neural network, two open medical datasets were selected for the experiment: (1) Lung Nodule Analysis—ISBI 2016 Challenge (LUNA16) [19] and (2) MICCAI-2017 Liver Tumor Segmentation Challenge (LiTS17) [20].

1. LUNA16, an international public dataset, is a subset of the largest publicly available pulmonary nodule baseline database, LIDC-IDRI [21]. This dataset contains a total of 888 chest CT images with 1,186 annotated pulmonary nodules larger than 3 mm in size. The slice thickness of all chest CT images was less than 2.5 mm, and the pixel resolution of each slice was 512×512. The pulmonary nodules in each image were annotated by four experienced radiologists.
2. The LiTS17 dataset contains 131 and 70 contrast-enhanced 3D abdominal CT scans for training and testing, respectively. The dataset was acquired by different scanners and protocols from six different clinical sites, with a largely varying in-plane resolution from 0.55 mm to 1.0 mm and slice spacing from 0.45 mm to 6.0 mm. The dataset provides ground truth for liver and liver tumours of 131 training data and 70 testing data.

The CT data provided by the above two data sets are three-dimensional data, and the training data used in this paper is two-dimensional data. we slice the z-axis to obtain the training image. 1186 lung nodule images and 201 liver tumor images are obtained from LUNA16 and LiTS17 respectively by the above method, and the obtained images are divided into training set and test set in an 8:2 manner. Therefore, the number of training sets and test sets of pulmonary nodules is 949 and 237 respectively. The number of training sets and test sets of liver tumors were 161 and 40 respectively.

### Methods

**Fig 1:**
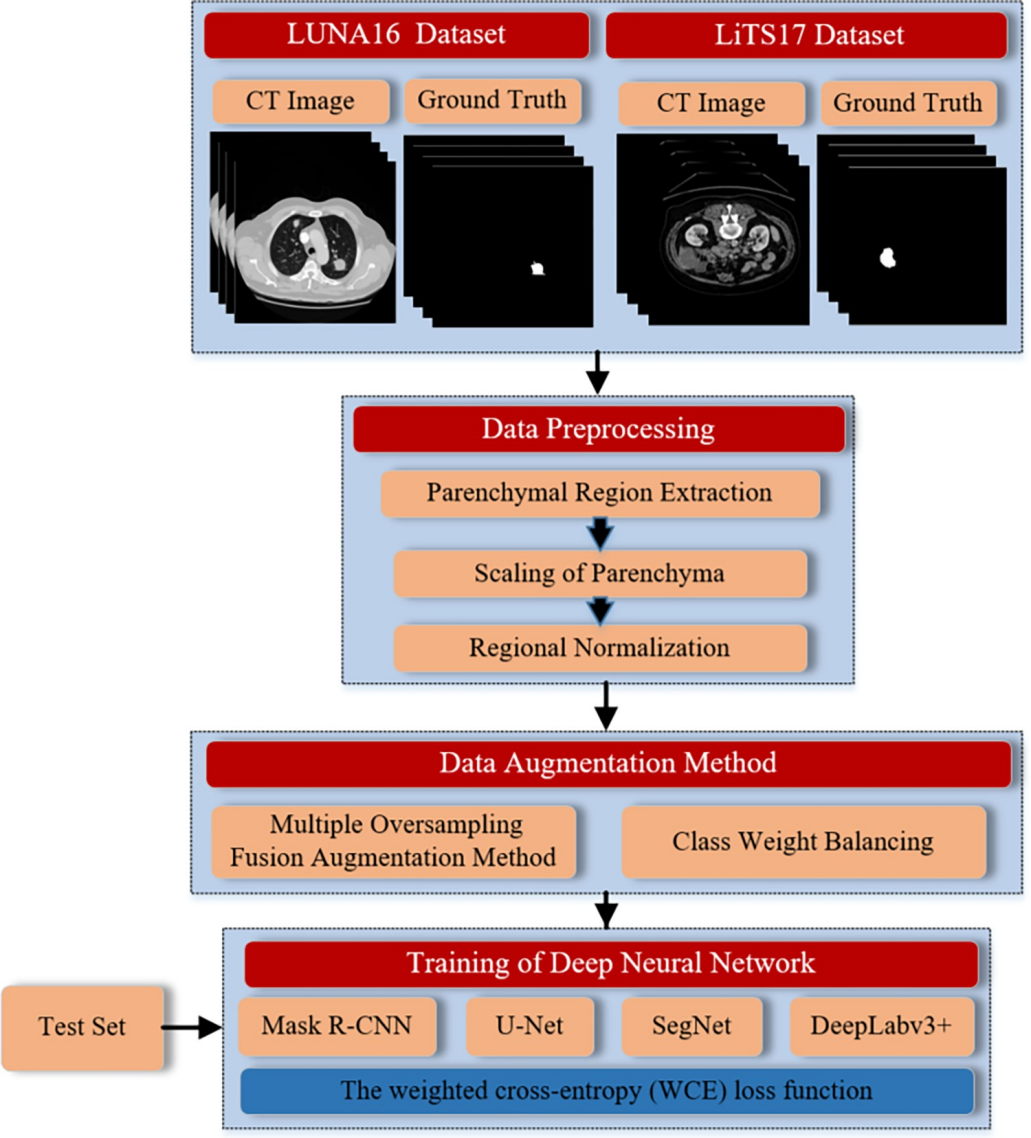
The framework of the proposed small tissue lesions segmentation method.

Fig 1 shows the framework of data augmentation based on multiple oversampling fusion for medical image segmentation. In this section, we introduce the data preprocessing and data augmentation methods in detail.

### Data preprocessing

**Fig 2:**
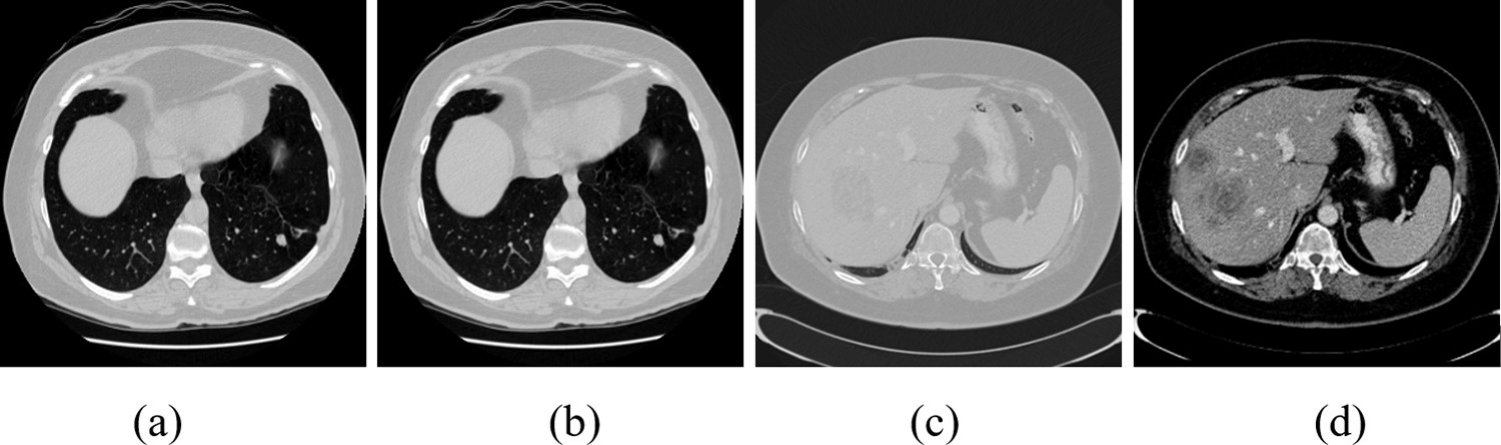
Samples of image after preprocessing. (a) Original image of LUNA16. (b) Fig (a) after preprocessing. (c) Original image of LiTS17. (d) Fig (c) after preprocessing.

For medical image volume, Hounsfield units (HU) are measurements of relative density determined by CT images. The HU value ranges from -1000 to 4069. Table 1 shows Hu values for common organs and typical objects. Because the surrounding bones, air or irrelevant tissues may interfere with the segmentation results, the initial segmentation method is used to filter these irrelevant objects to keep the corresponding tissues clean and segmented. In radiological imaging, such as CT, a window width filter is often used to remove the HU value of unnecessary tissues. The main steps are as follows: Firstly, we set the Hu value in the corresponding interval of different tissues. The window width of lung tissue is set to [–1000,250], and that of liver tissue is set to [–100,250]. Most of the unrelated tissues and organs were removed after treatment, as shown in Fig 2. Secondly, due to the relatively fixed position of human tissues, according to the ground truth of lung parenchyma and liver tissue regions provided by the dataset, the smallest external square was used to intercept the corresponding tissues and enlarge their size to 512×512. Then the images were normalized to ensure that the pixel values range from 0 to 1.

**Table 1:** HU values of typical objects and organs.

objects	HU values
bone	>400
blood	7~32
liver	40~70
water	0±5
air	-1000

### Augmentation method

**Fig 3:**
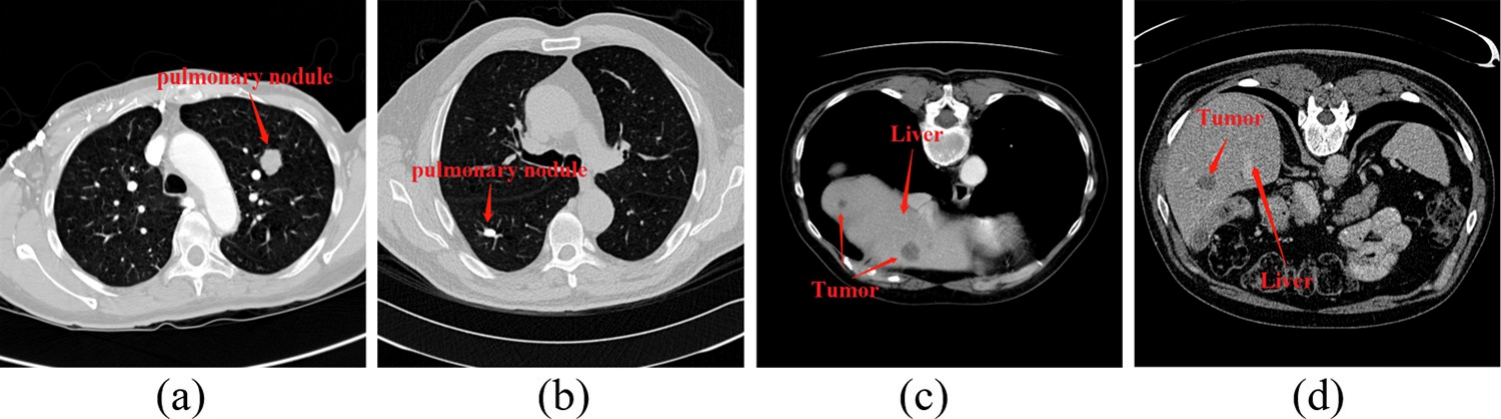
Distribution of tissue lesions in CT images. (a) and (b) are pulmonary nodules, and (c) and (d) are liver tumours.

In CT images, tissue lesion areas only account for a small part of the overall image. The background pixels of the image are much larger than the pixels of the tissue lesion, resulting in a serious imbalance in the category, as shown in Fig 3. For example, in LUNA16, the ratio of pixels for background and pulmonary nodules, which is less than 3 mm, is only 98.2:1.8, and in the 20 mm pulmonary nodules, the ratio of pixels for background to pulmonary nodules is 93.3:6.7. However, in LiTS17, the ratio of background to liver tumour is 89.2:10.8. This kind of category imbalance is not conducive to network learning, so the network learns more categories of information, resulting in the final discrimination results being biased towards categories.

#### Oversampling

**Fig 4:**
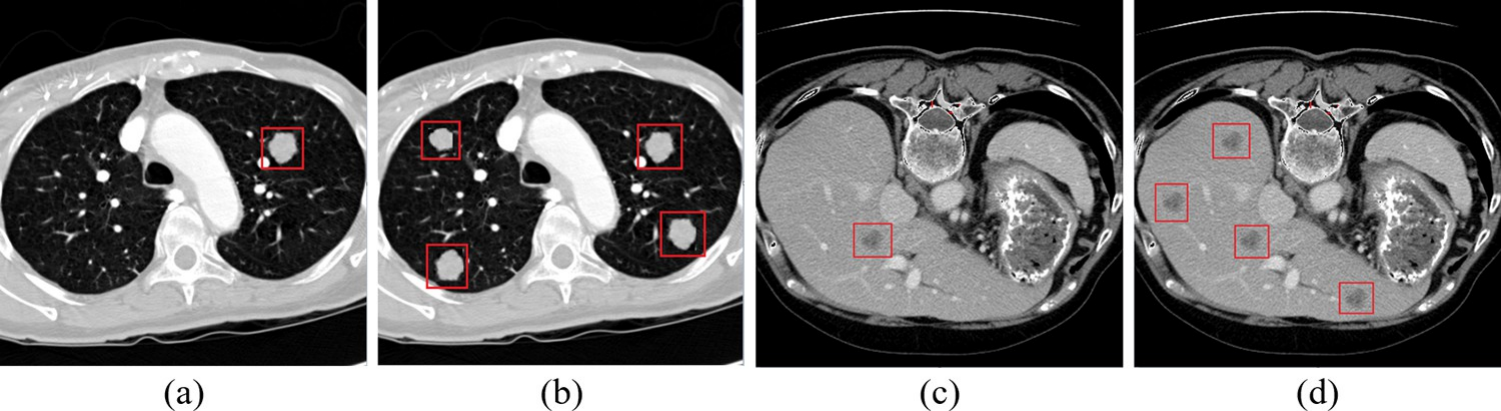
Examples of oversampling. (a) and (c) show the original images of pulmonary nodules, while (b) and (d) show postsampling examples corresponding to images (a) and (c) the sampling rate in these examples was set to 4.

To address the imbalanced class problem in datasets, a method of randomly oversampling the tissue lesion samples within a single image was proposed, and the method improved both the number of samples and the pixel proportion of tissue lesions in the image. The specific implementation process is as follows: a segmentation mask of tissue lesions obtained by the previous label is constructed using the original tissue lesion locations and combined with the segmentation mask of the organ region provided by the dataset. This ensures that the tissue lesions will be oversampled within the organ region. Additionally, it ensures that new samples do not overlap with any existing samples and are at least 5 pixels away from the image boundary. Some examples of LUNA16 and LiTS17 are shown in Fig 4. Different from the oversampling where a new object is obtained by synthesizing two different lesion regions, the proposed random oversampling strategy aims to increase the number of lesion regions in the image using a similar repeated sampling original sample mode.

#### Geometric transformation:

Geometric transformation of image is the most commonly used data expansion method in image classification and segmentation tasks. It can effectively increase the diversity of samples in the training set. It is an effective method to solve the overfitting of network models. The geometric transformation of images includes translation, rotation, scaling, mirroring, etc. Suppose *i* and *j* are coordinates in the image *S*, the rotation and mirror image can be expressed by the Formulas (1) and (2), respectively:

$$\{\begin{array}{c} i^{\prime }=icos\theta ‐jsin\theta \\ j^{\prime }=isin\theta +jcos\theta \end{array}$$ (1)

$$\{\begin{array}{c} i^{\prime }=i\\ j^{\prime }=N‐j+1 \end{array}or\;\{\begin{array}{c} i^{\prime }=M‐i+1\\ j^{\prime }=j \end{array}$$ (2)

Where *M* and *N* denote the width and height of the image *S*, respectively. The scaling of the image needs to be realized by reducing or increasing the pixels of the image. Image reduction will reduce the number of pixels in the image, and it is usually necessary to sample the image to ensure that the image will not be distorted. On the contrary, the enlargement of the image will increase the pixels of the image, and the increased pixels will be filled by the difference.

#### Multiple oversampling fusion

**Fig 5:**
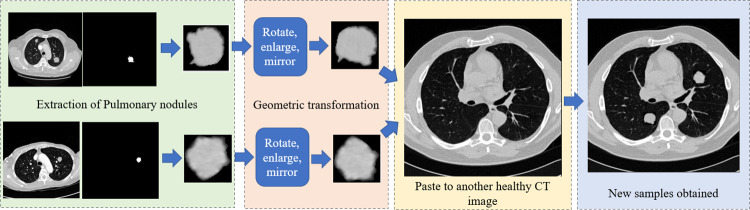
Specific process of multiple oversampling fusion augmentation method.

**Fig 6:**
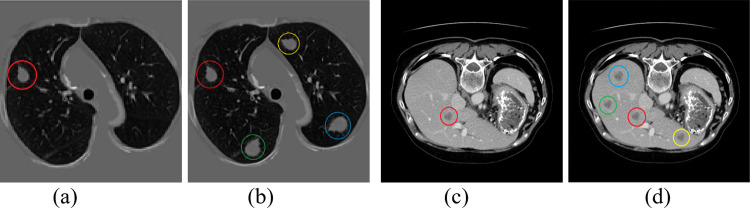
Samples of multiple oversampling fusion augmentation method. (a) original image of pulmonary nodule image, (b) is the result of (a) using fusion oversampling, (c) original image of liver tumor image, and (d) is the result of (c) using fusion oversampling. (a red circle represents the original lesion area, a yellow circle represents mirrored and rotated lesion area, a blue circle represents magnified and rotated lesion area, and a green circle represents magnified and mirrored lesion area).

To address the imbalanced class problem in datasets, a multiple oversampling fusion augmentation Method is proposed, which improves the number of samples and pixel ratio of tissue damage in the image. The specific process is shown in Fig 5. Using the original tissue damage location and combining with the segmentation mask of the organ region provided by the dataset, the segmentation mask of the tissue damage obtained by the previous tag is constructed. The extracted tissue lesion region is used. This ensures that the sampled object conforms to the prior knowledge, that is, the tissue lesion must be located on the corresponding tissue. Therefore, we randomly paste the sampled object into the tissue and ensure that it is at least 5 pixels away from the image boundary. In order to increase the diversity of samples, we randomly rotate, enlarge and mirror the sampled objects before placing them in the tissue area. Using this method, the original lesion images from the training set are used to increase the total number of lesion objects in the training set, but not increase the total number of training images. Some examples of LUNA16 and LiTS17 are shown in Fig 6. The concrete implementation of the proposed data augmentation method is described in Algorithm 1.

**Algorithm 1** Multiple Oversampling Fusion augmentation method.

**INPUT:** Dataset *D*, Image without lesions *I*, area of organ *A*, Sampling ratio *m*

**OUTPUT:** synthetically generated image I*

1: **for** *i* < *m* **do**

2: *S*_*i*_ = Random get lesion sample for *D*

3: *θ* = Rand (0, *π*)

4: *H*_*i*_ = Rotate (*S*_*i*_, *θ*)

5: *ω* = Rand (0.5, 2.0)

6: *M*_*i*_ = Enlarge (*H*_*i*_, *ω*)

7: *K*_*i*_ = Mirror (*M*_*i*_, *τ*) #*τ* represents horizontal mirror or vertical mirror

8: (*w*, *h*) = Random coordinates in the image *I*

9: **if** (*w*, *h*) ∈ *A* **then**

10: *U*_*i*_ = (*w*, *h*, *w*+*x*, *h*+*y*) #*x*, *y* represent the width and height of *K*_*i*_, respectively

**end**

11: I* = I*∪*U*_*i*_

12: **end for**

13: **return** I*

#### Class weight balancing

Through the multiple oversampling fusion augmentation method, the class imbalance in the data set can be improved to a certain extent, but the pixels of the background in the image are still much higher than the pixels of the lesion object. In order to further offset the imbalance of categories in the data set, we assign a larger weight to the labels with a smaller total number and a smaller weight to the categories with a larger total number.

If the number of pixels in a particular class is denoted as *N*_*c*_, where c corresponds to background and lesion. Hence, *N*_*c*_ is (1×2) array. Let *T* represent the total number of pixels in the images, then the (1×2) array of image frequency, *F*_*c*_, of a class is the ratio of *N*_*c*_ to *T* given by:

$$F_{c}=\frac{N_{c}}{T}$$ (3)

From here, the (1×2)-array of class weight, *Wc*, for a set of training data can be calculated by finding the ratio of median of *F*_*c*_ to *F*_*c*_:

$$W_{c}=\frac{median(F_{c})}{F_{c}}$$ (4)

**Fig 7:**
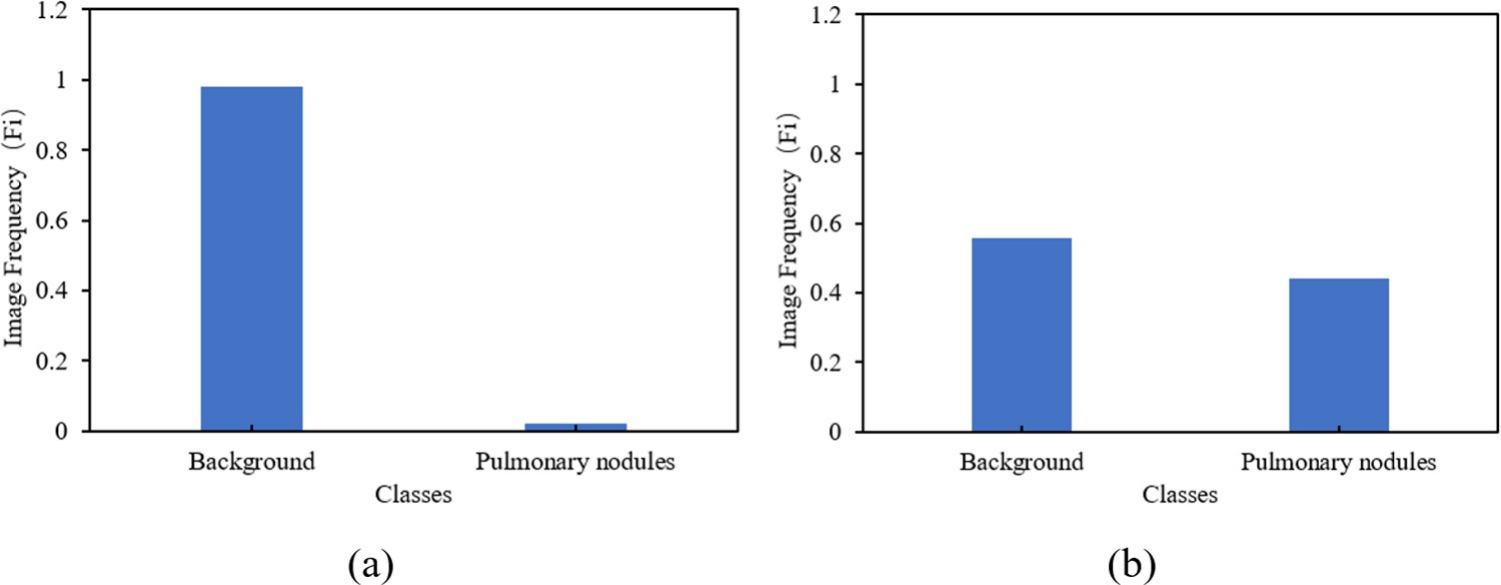
Pixel distribution of (a) original images and (b) after class weighting for the LUNA16 dataset.

**Fig 8:**
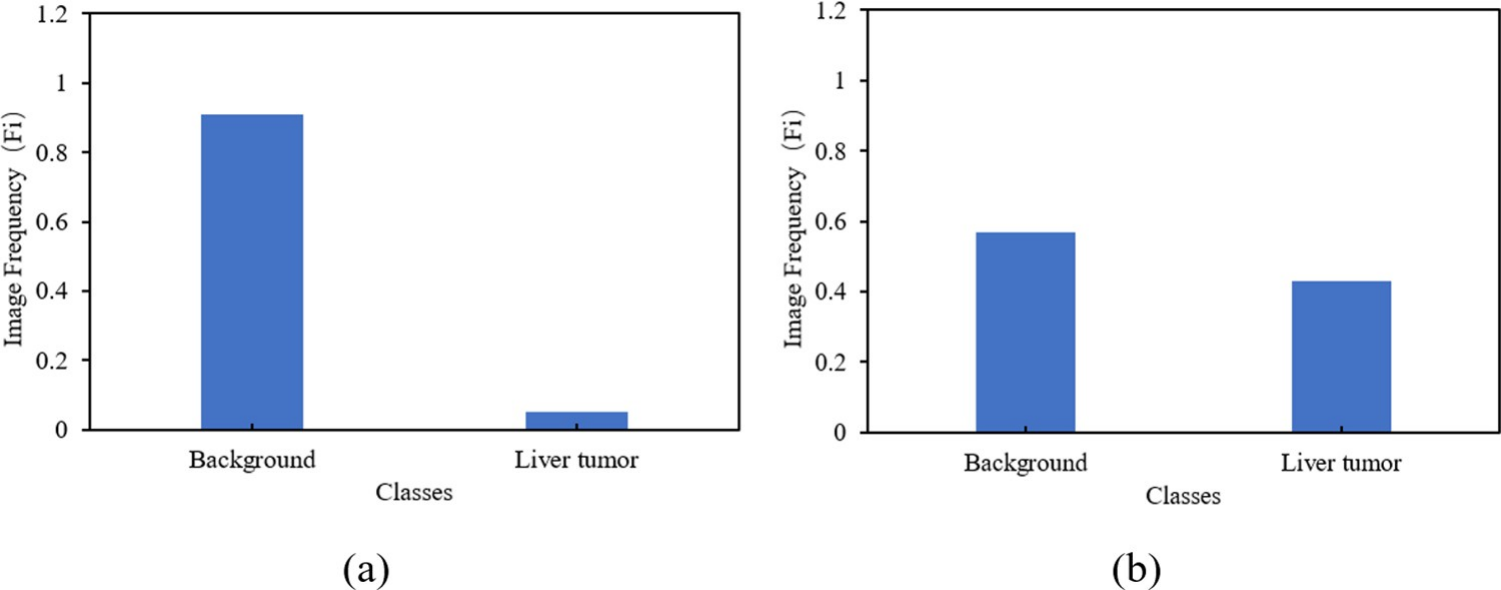
Pixel distribution of (a) original images and (b) after class weighting for the LiTS17 dataset.

Histograms of pixel distribution of LUNA16 and LiTS17 dataset, before and after applying class weighting approach, are shown respectively in Figs 7 and 8.

### Network architecture and loss function

#### Network architectures

We selected four segmentation models to evaluate the comparative performance in medical image segmentation: Mask-RCNN, U-Net, SegNet and DeepLabv3+. These four models were selected because they have yielded excellent performance in other applications, including road scenes, biomedical images, and natural image segmentation. Each deep neural network is briefly described below.

**Fig 9:**
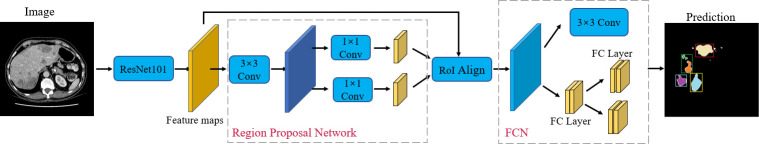
The detailed architecture of the Mask-RCNN.

Mask-RCNN [22] was developed based on the Faster RCNN model and adds an ROI Align layer and a fully convolutional network (FCN) [23]. Mask-RCNN splits classification prediction and mask prediction into two network branches. Each binary mask produced by the mask prediction branch depends on the classification prediction result based on the separation of objects at that moment. Mask-RCNN uses the ROI Align layer to uniformly define the ROI size and then inputs it into the two classifier branches. The Faster RCNN network is used for category and position prediction, while the FCN network is used for pixel-level segmentation. The specific architecture of Mask-RCNN is shown in Fig 9.

**Fig 10:**
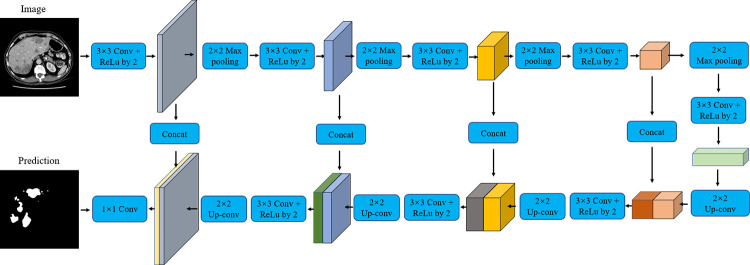
The detailed architecture of the U-Net.

U-Net [24] has been widely used in medical image segmentation since it was proposed. This is because it can work and produce better segmentation performance when few training images are available. The network is composed of an encoder decoder network, and each layer is connected by skip connections. The encoder uses a convolution layer to extract feature maps from the input image, while the decoder performs upsampling to recover the image resolution from the encoder feature maps. The whole architecture consists of four convolution layers. After each downsampling, the number of filters is doubled. Correspondingly, the upsampling stage and the two convolution operations are repeated four times; in each stage, the number of filters is halved. Before the merging operation, the feature mapping information from the convolution operation of the encoder is transmitted to the decoder. Skip connections between encoder and decoder networks help to recover information lost during pool operations. Finally, the final segmentation result is obtained by 1 × 1 convolution. The specific architecture of U-Net is shown in Fig 10.

**Fig 11:**
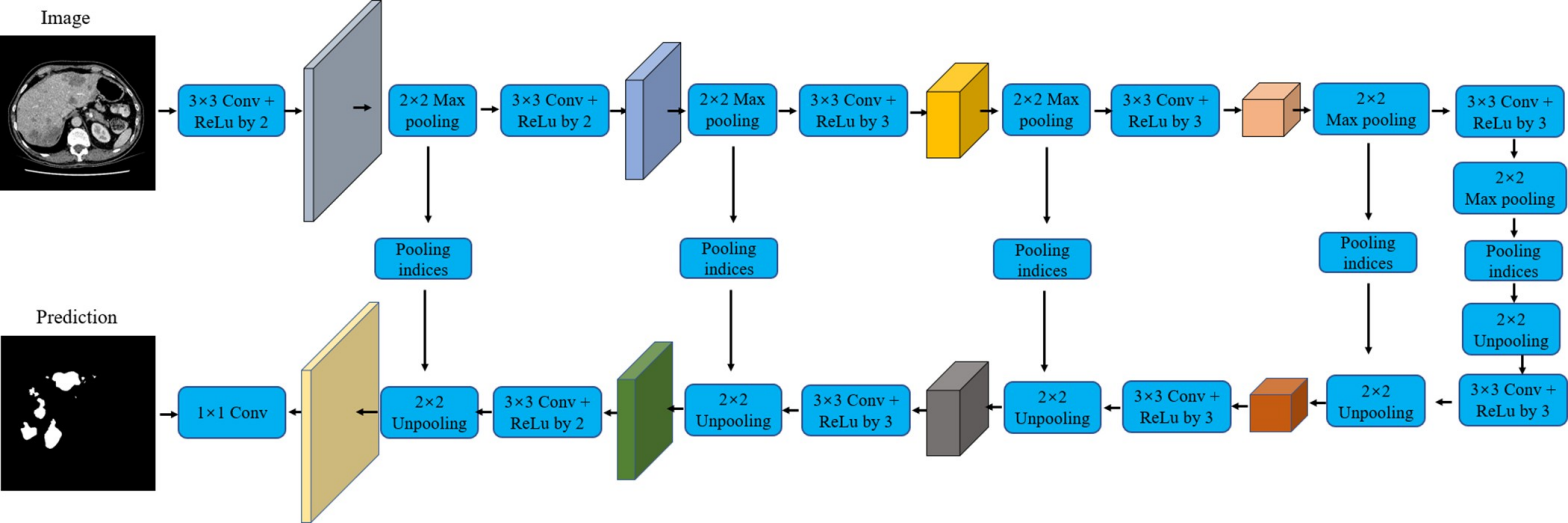
The detailed architecture of the SegNet.

SegNet [25] is a pixelwise segmentation technique that was first developed for outdoor and indoor scene understanding. The network architecture is composed of a forward connected encoder based on a visual geometry group architecture, an untrained layer, a group of corresponding decoders and a pixel-level classifier. To make the model suitable for efficient embedded systems, the designers removed the full connection layer and reduced the network parameters from 134M to 14.7M. The maximum pooling and subsampling operations reduce the resolution of feature mapping and output, which leads to the poor performance of the network applied to pixel segmentation. To solve the problem that the resolution of the input image is different from that of the output image, SegNet uses the stored pool index to upsample the low-resolution feature map. This not only improves the ability of the network to obtain boundary information but also reduces the number of parameters that must be trained. The specific architecture of SegNet is shown in Fig 11.

**Fig 12:**
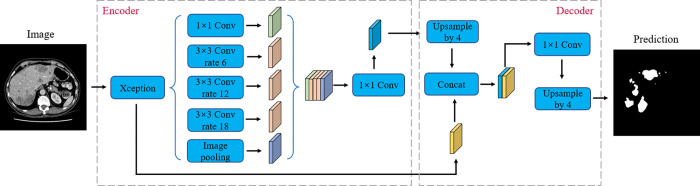
The detailed architecture of the DeepLabV3+.

DeepLabV3+ [26] is a semantic segmentation network with encoder–decoder. This architecture is characterized by the use of atrous or dilated convolution and atrous spatial pyramid pooling (ASPP). DeepLabV3+ used the Xception model as the backbone and replaced the maximum pooling layer with a depthwise separable convolution to maintain the spatial resolution of the output feature map. In contrast to using standard convolution, deep separable convolution is used, which divides the operation into depthwise convolution and point convolution. Feature maps are obtained by applying depthwise convolution to each input channel, which performs separable convolution using zeros placed in continuous filters. The output of depthwise convolution is accumulated by utilizing pointwise convolution. The specific architecture of DeepLabV3+ is shown in Fig 12.

### Loss function

It is common for tissue lesions to occupy only a very small area in medical images. This usually leads to the learning process falling into the local minimum of the loss function, resulting in a network whose prediction is strongly biased towards the background. We adopted the weighted cross-entropy (WCE) loss function for the CNNs because this loss assists the network in differentiating between background and tissue lesions. The WCE loss function penalizes each class based on its median frequency, which is formulated as follows:

$$WCE=‐\frac{1}{n}\sum_{i=1}^{n}W_{c,i}[T_{i}logP_{i}+(1‐T_{i})log(1‐P_{i})]$$ (5)

This sum is executed for all the training images, *n*. The variable *P*_*i*_ is the predicted segmentation class, *T*_*i*_ is the target or the ground truth segmentation label, and *W*_*c*,*i*_ is the class weight calculated from Eq (4).

### Evaluation metrics

Precision (P), Recall (R), Dice similarity coefficient (Dice) and Volumetric Overlap Error (VOE) metrics are used to evaluate the segmentation performance of the different models. DSC is a widely used metric for measuring the overlap between two segmentation results. VOE indicates the error rate of the segmentation result. They are defined as follows:

$$P=\frac{TP}{TP+FP}$$ (6)

$$R=\frac{TP}{TP+FN}$$ (7)

$$Dice=\frac{2TP}{2TP+FP+FN}$$ (8)

$$VOE=1‐\frac{TP}{TP+FP+FN}$$ (9)

where *TP*, *FP* and *FN* represent the number of true positive, false positive, and false negative samples, respectively. In order to verify the performance of each algorithm, we used 10-fold cross validation to train and test the model. All the following results are presented as mean ± standard deviation (mean ± std).

## Experiments and results

### Experimental parameter settings

For this experiment, we developed the code for our method using Keras, and the models were implemented in Python. The models were executed on a computer equipped with an Ubuntu 16.04 operating system, 32 GB of memory, an Intel Core i7-8700k (3.7 GHz) CPU, and a GeForce RTX 2080ti GPU (with CUDA 10.1 and 11 GB of memory). We selected the Adam optimizer as the network optimization algorithm. The learning rate was set to 0.0001. The minimum batch size was set to 2. The number of training iterations for each network was limited to 5000. The experiment assesses the influence of different data augmentation methods on the four networks and uses the test results of each network without data augmentation as the reference value for the corresponding network.

### Augmentation technique analysis

To verify the influence of the data augmentation method proposed in this paper on the performance of network segmentation. We compared the segmentation performance of the four networks with and without data augmentation training on LUNA16 and LiTS17, and the results are shown in Tables 2 and 3, respectively. no-aug, mof-aug represent no augmentation and multiple oversampling fusion augmentation respectively. We used a 10-fold stratified cross validation strategy to test all algorithms, in which each image appeared once in the test set over all folds. All results are multiplied by 1000 and the bold font highlights the best results. Tables 2 and 3 shows the results of segmentation performance of all algorithms. The results show that the multiple oversampling fusion augmentation technique is capable of segmenting lesion area with high performance compared to the other techniques.

**Table 2:** Performance of pulmonary nodules segmentation on LUNA16 data sets.

Models	Methods	LUNA16			
		P	R	Dice	VOE
**Mask-RCNN**	no-aug	745±68	664±61	702±70	459±59
**U-Net**		731±54	790±51	759±50	388±60
**DeepLabV3+**		755±55	693±57	723±71	434±58
**SegNet**		725±58	752±63	738±56	415±61
**Mask-RCNN**	mof-aug	806±51	**854±49**	**829±45**	**292±43**
**U-Net**		819±53	806±51	812±48	316±45
**DeepLabV3+**		**830±49**	769±56	798±52	336±51
**SegNet**		803±53	831±59	817±55	309±49

**Table 3:** Performance of liver tumor segmentation on LiTS17 data sets.

Models	Methods	LiTS17			
		P	R	Dice	VOE
**Mask-RCNN**	no-aug	754±65	717±58	735±68	419±61
**U-Net**		747±60	786±55	766±51	379±59
**DeepLabV3+**		711±54	789±56	748±67	403±63
**SegNet**		760±63	726±61	743±65	409±57
**Mask-RCNN**	mof-aug	859±48	**901±51**	**879±40**	**215±39**
**U-Net**		**861±50**	824±50	842±44	273±42
**DeepLabV3+**		827±47	839±53	833±47	286±48
**SegNet**		852±49	805±55	828±45	294±50

### Results of calculation efficiency

The running time of implementing different methods on the testing data is shown in Table 4. It can be seen from the table that the running speed of U-Net is the fastest, and its running speed can reach 1.53 fps. Next are SegNet, Mask-RCNN and DeepLabV3+, whose running speeds are 1.42 fps, 1.24 fps and 1.09 fps, respectively.

**Table 4:** The running time of different methods in segmenting lesion.

Models	Parameters	FPS(fps)
**Mask-RCNN**	39.1M	1.24
**U-Net**	28.8M	1.53
**DeepLabV3+**	41M	1.09
**SegNet**	29.4M	1.42

### Comparison with other methods

To illustrate the effectiveness of this method, we compared the results with other methods. we used the best performing model from Tables 2 and 3, namely the Mask RCNN model. We tested three different training configurations of Mask RCNN to evaluate their performance in more detail: (1) training using translate, rotate and dilate data augmentation(t-aug), (2) Samplepairing data augmentation (sp-aug) [27], (3) mixup data augmentation (mix-aug) [28], (4) training using multiple oversampling fusion augmentation(mof-aug). For fairness of comparison, we expanded the number of images in the training set of each method to the same number. We used a 10-fold stratified cross validation strategy to test all algorithms, in which each image appeared once in the test set over all folds. The results are shown in Table 5. All results are multiplied by 1000 and the bold font highlights the best results. According to the experimental results shown in Table 5, the proposed method is superior to the existing augmentation methods.

**Table 5:** Performance of lesion segmentation on LUNA16 and LiTS17 data sets.

Models	LUNA16				LiTS17			
	P	R	Dice	VOE	P	R	Dice	VOE
**Mask-RCNN t-aug**	698±59	783±61	738±64	415±51	785±64	781±63	783±68	357±56
**Mask-RCNN sp-aug**	792±53	736±52	763±57	383±47	805±55	844±53	824±53	299±44
**Mask-RCNN mix-aug**	758±55	795±57	776±60	366±49	854±53	826±57	837±55	276±43
**Mask-RCNN mof-aug**	806±51	**854±49**	**829±45**	**292±43**	859±48	**901±51**	**879±40**	**215±39**

**Fig 13:**
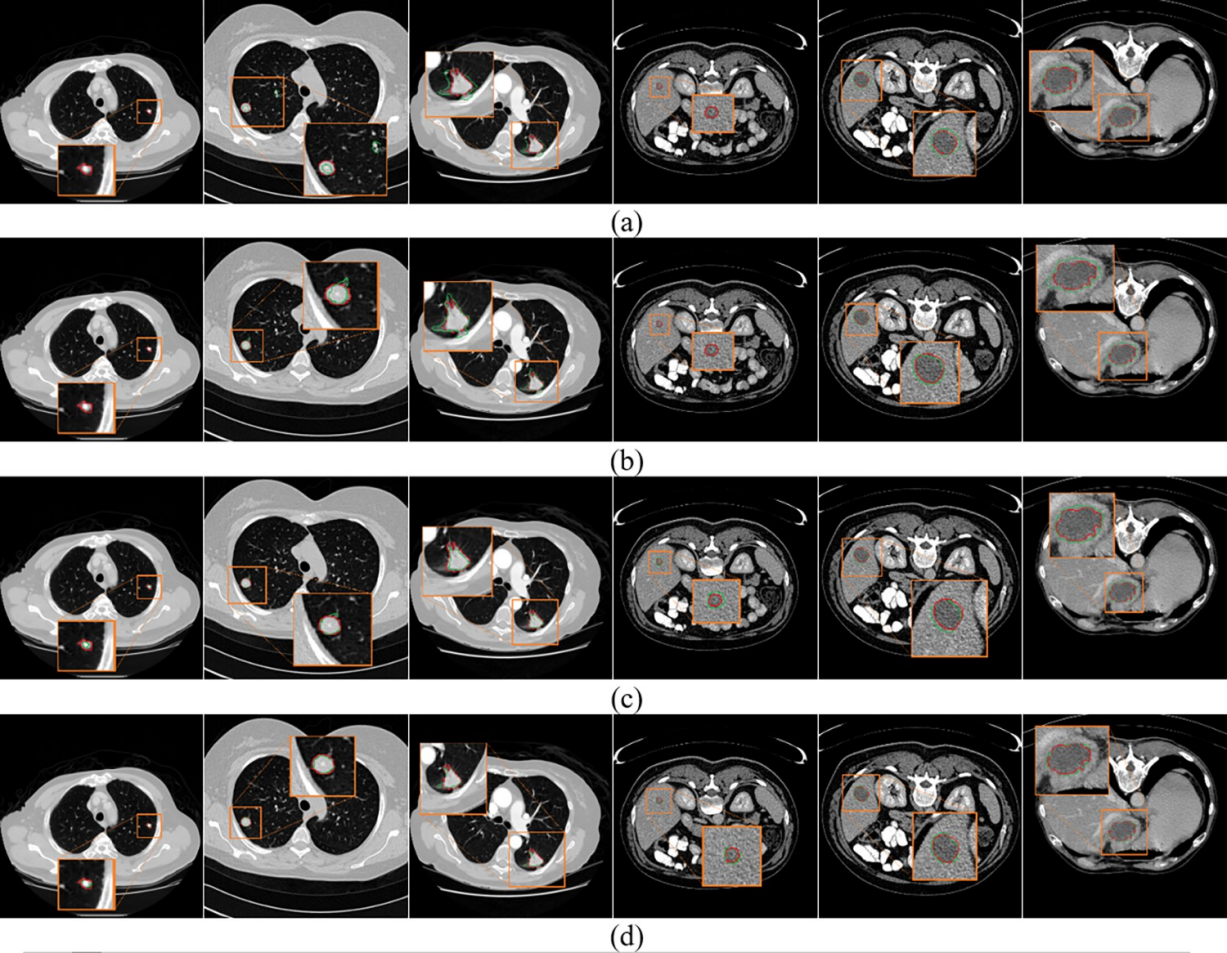
A visual comparison of the lesion segmentation results. (a)t-aug. (b)sp-aug. (c)mix-aug. (d)mof-aug. The red and green contours denote the ground truth and the segmentation results, respectively.

To allow a visual comparison of different approaches, the segmentation results are given in Fig 13. We show the segmentation results of different size lesions on LUNA16 and LiTS17 test sets for visual comparison. For large-scale objects, all methods can ensure good segmentation accuracy. However, when segmenting small-sized lesions, t-aug and ap-aug may miss segmentation, and method 1 may recognize pulmonary vessels as pulmonary nodules. The implementation of Samplepairing is very simple. The pixels of two pictures are added directly to average, and the supervised label is unchanged. This increases data diversity and also introduces noise. So the lifting effect of this method is very limited. Mix-aug can recognize and segment it, but there is a large gap between the segmentation accuracy and the real value. This method can accurately identify small target objects, and the segmentation accuracy is obviously better than that of mix-aug.

### Performance comparison with state-of-the-art

To illustrate the efficiency of the proposed method, we compared the results with those of other methods. Two different comparisons are provided: (1) We compared the network with the best segmentation performance (Mask-RCNN) achieved with the recently proposed segmentation methods. (2) We selected the network with the best performance at present to expand the training data with the method proposed by us and compared the impact on network performance before and after dataset augmentation.

Tables 6 and 7 show the segmentation performance of other methods and our work on pulmonary nodules and liver tumours, respectively. Our work cannot be directly compared with other methods due to the different datasets, pretreatment methods and training data volumes used. If we only consider the final segmentation result (DSC value), the best performance in pulmonary nodule segmentation was demonstrated by CoLe-CNN, proposed by Pezzano et al. [29] with a DSC value of 0.829±0.054. The network with the best liver tumour segmentation performance was MS-UNET proposed by Kushnure et al. [30], with a DSC value of 0. 889±0.051 Mask-RCNN trained by using the proposed method can achieve 0.829±0.045 and 0.879±0.04 in the segmentation of pulmonary nodules and liver tumours, respectively. By comparison, the segmentation performance of Mask-RCNN trained by the proposed method after data augmentation is comparable to that of the existing optimal segmentation network. It can be seen from the above that the best lesion region segmentation effect is obtained on mask RCNN using the data augmentation method proposed in this paper.

**Table 6:** Comparison of our work to pulmonary nodule segmentation state-of-the-art methods. All results are multiplied by 1000 and the bold font highlights the best results.

Work	Year	Dataset	Network	DSC
Wang et al. [9]	2017	LIDC-IDRI	CF-CNN	793±91
Shen et al. [31]	2017	LIDC-IDRI	MC-CNN	788±82
Sun et al. [32]	2017	LIDC-IDRI	MCROI-CNN	802±74
Havaei et al. [3]	2017	LUNA16	Cascaded-CNN	818±78
Cao et al. [33]	2020	LIDC-IDRI	DB-ResNet	827±62
Pezzano et al. [29]	2021	LUNA16	CoLe-CNN	829±54
**Our work**		**LUNA16**	**Mask-RCNN**	**829±45**

**Table 7:** Comparison of our work to liver tumour segmentation state-of-the-art methods. All results are multiplied by 1000 and the bold font highlights the best results.

Work	Year	Dataset	Network	DSC
Jin et al.[11]	2020	LiTS17	RA-UNet	719±81
Qin et al.[34]	2018	LiTS17	SBBS-CNN	740±73
Li et al.[12]	2018	LiTS17	H-DenseUNet	831±53
Seo et al. [35]	2020	LiTS17	Modified U-Net	856±48
**Kushnure et al. [30]**	**2021**	**LiTS17**	**MS-UNet**	**889±51**
Our work		LiTS17	Mask-RCNN	879±40

## Discussion

Automatic lesions segmentation plays an important role in cancer diagnosis. It provides the precise contour of the lesions inside the anatomical segments of the organ, which assists doctors in the diagnosis process. However, lack of training data and imbalance of classes are common problems of medical data. In this paper, a multiple oversampling fusion data augmentation method be proposed to solve the problem of data shortage and classes imbalance. With a variety of synthetic data generation techniques we use the lesion region of the original data to augment the data for training the deep neural network. Benefiting from data generation strategy of oversampling we showcased the efficiency of the technique in comparison with other data augmentation.

**Fig 14:**
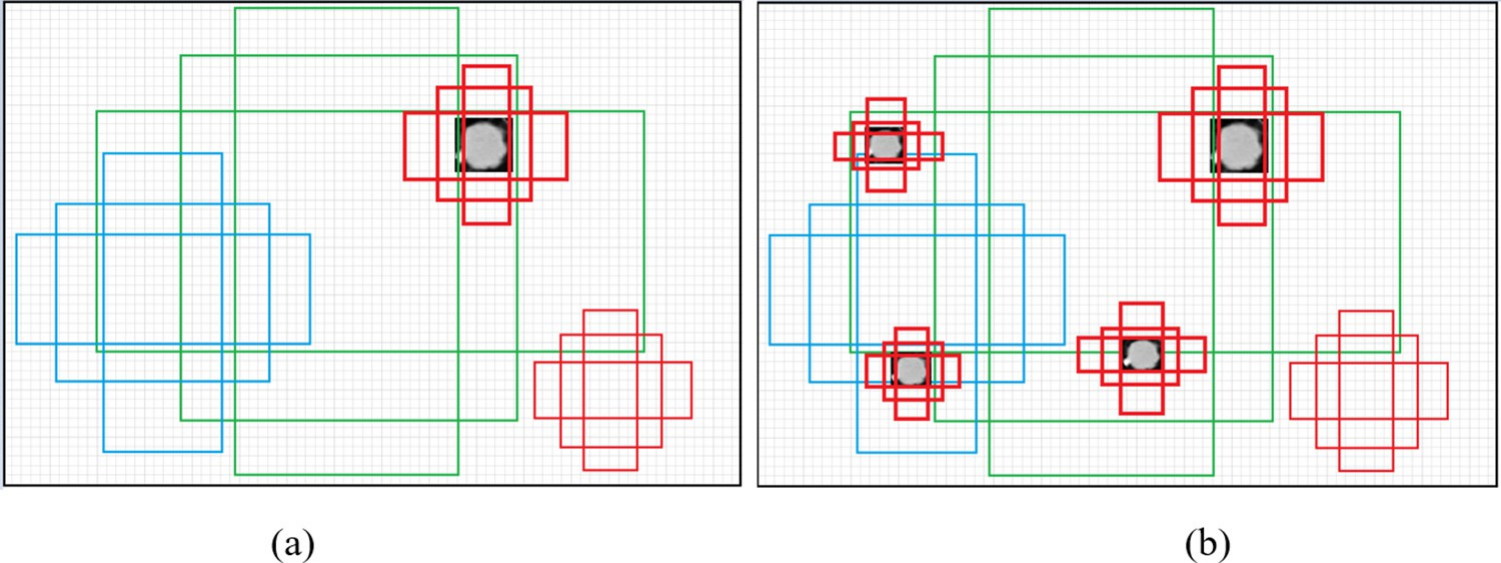
Schematic diagram of anchor frame matching with pulmonary nodules. (a) Anchor frames that matched images of pulmonary nodules in the baseline training set. (b) Anchor frames that matched images of pulmonary nodules in the training set after random oversampling.

To show the generalization capability of our method in the clinical practice. We compare the segmentation performance before and after using our proposed data augmentation on four common deep convolution neural networks. The results show that the segmentation accuracy of the trained network model is improved after the data augmentation on LUNA16 and LiTS17. There may be two reasons for this. First, the integrated oversampling data augmentation method is helpful to improve the imbalance classification of the dataset, which makes the decision threshold shift to the major category and improves the discriminant ability of the network. Second, oversampling and affine transformation increase the diversity of training data. They can also change the class distribution and sample number of the dataset. As shown in Tables 2 and 3, the best segmentation performance is obtained on Mask-RCNN, whether LUNA16 or LiTS17. This is closely related to the network structure and training strategy of Mask-RCNN. Mask-RCNN is composed of two parts: a regional suggestion network and an ordinary CNN network. In practice, it is easy to miss smaller tissue lesions in the process of identification and matching, so the performance of the whole network is affected. The multiple oversampling fusion augmentation method increases the number of samples in the same image, which can effectively reduce the probability of missing small target objects in the process of network training. In the training process, more anchors are matched with the training samples (as shown in Fig 14), thus improving the network segmentation performance for small target objects [17].

**Fig 15:**
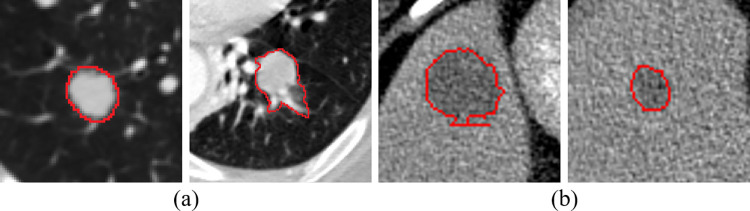
The images of lung nodules and liver tumors. (a) images of lung nodules. (b) Liver tumor image.

It can be seen from Tables 2 and 3 that the proposed data augmentation method achieves different improvement effects on LUNA16 and LiTS17 dataset. Probably because the morphological diversity and complexity of lung nodules is significantly higher than that of liver tumours, as shown in Fig 15. It might be hard to guarantee a full coverage of all possible types of lung nodules. Meanwhile, most of the liver tumours were larger in size than those of the lung nodes. We analyze the effectiveness of the proposed method for different size lesions. The results are shown in Table 8. We can observe that the proposed method obtains a better performance improvement for the large lesions than the small lesions. The number of small lung nodules in LUNA16 dataset was significantly higher than that of small liver tumours. Therefore, the performance of the network on LiTS17 dataset will be better than that on LUNA16 dataset. In the future, we will focus on the segmentation for small lesions. Recently, generative adversary network (GAN) has been proposed for small object detection and classification. For example, Li et al. [36] generated a super-resolution representation of small objects by finding the intrinsic structural correlation between small-scale and large-scale objects, which may also be a potential direction to deal with this challenging problem.

**Table 8:** Effectiveness of Mask RCNN to the lesion size (Dice:%).

	LUNA16		LiST17	
	Small-nodules	Large-nodules	Smal-tumor	Large-nodules
number	754	432	63	138
no-aug	68.1	75.2	70.5	75.2
mof-aug	77.6(+9.5)	89.4(+14.2)	81.6(+11.1)	90.5(15.3)

## Conclusions

In this study, we propose a multidimensional data augmentation method that combines affine transform and random oversampling strategy to address the segmentation problem of unbalanced data distributions. Our main conclusions are as follows:

1. Compared with the other data augmentation method, the multiple fusion oversampling data augmentation method proposed in this paper has a good effect on small target segmentation in the case of unbalanced sample distribution. The experiments on the common datasets LUNA16 and LiTS17 further prove that the proposed data augmentation method can effectively improve the performance of different network models in tissue damage segmentation. The best pixelwise segmentation performance for both pulmonary nodules and liver tumours was obtained by the Mask-RCNN model, with DSC values of 0.829 and 0.879, respectively.
2. Compared with the latest CNN based technologies such as MS-Unet, CoLe-CNN and Modified U-net, it is proved that the Mask-RCNN trained through the multiple fusion oversampling data augmentation method and class weight balancing has comparable performance with them in lesion region segmentation. This can be attributed to its network combination and Region Proposal strategy.
